# Contested illness and communication: Positioning long Covid in online discussion forums

**DOI:** 10.1177/13591053251389517

**Published:** 2025-11-13

**Authors:** Sally Sargeant, Sarah Wilkinson, Mimirose Lorraway, Suzanne McDonald

**Affiliations:** 1 Faculty of Health, Southern Cross University, Coolangatta, Queensland, Australia; 2 Department of Communities and Justice, Lismore, NSW, Australia; 3 Medical School, University of Queensland, Herston, Queensland, Australia

**Keywords:** long COVID, sick role theory, positioning theory, online forums, contested illness

## Abstract

The World Health Organization (WHO) officially acknowledged long COVID as a post COVID-19 condition in 2021. Emerging research shows that long COVID may be considered a contested illness due to unknown causal mechanisms, diagnostic uncertainty, poor recovery/treatment outcomes and people reporting illness dismissal experiences when seeking healthcare. Informed by the sick role and positioning theories, this study explored people’s experiences of seeking healthcare for long COVID by examining posts in two online forums (Mayo Clinic Connect and Reddit, from June and July 2023). Template analysis helped to identify12 forum threads containing a total of 305 posts. Inductive thematic analysis of posts identified three themes: (a) ‘The concept of time’, (b) ‘The importance of symptom legitimacy’, and (c) ‘Responding to diagnostic dismissal’. Healthcare professionals are critical in validating the patient experience without a formal long COVID diagnosis, and in supporting long COVID recovery needs by acknowledging heterogeneity and diagnostic difficulties.

## Background

Early in the SARS-CoV-2 (COVID-19) pandemic, public health messaging presented COVID-19 as a mild acute infection with an expected four-week recovery time (Barker et al., 2022). However, people began to share their experiences of poor recovery outcomes within online platforms (Callard and Perego, 2021; Miyake and Martin, 2021; Rushforth et al., 2021). These shared experiences led to the widely recognised term ‘long COVID’ (Perego et al., 2020; Roth and Gadebusch-Bondio, 2022). Though clinically defined, long COVID continues to have undetermined causal links, poor recovery/treatment outcomes and diagnostic uncertainty (Altmann et al., 2023). Long COVID is argued to align with illnesses deemed to be contested, such as myalgic encephalomyelitis/chronic fatigue syndrome (ME/CFS) (Au et al., 2022; Barker et al., 2022; Neaves, 2022) due to the continued diagnostic challenges experienced by patients and healthcare professionals (Dumit, 2006).

Contested illnesses are particularly associated with diagnostic uncertainty, poor clinical knowledge of the causal mechanisms that induce symptom onset, and patients often experience illness dismissal (Barker et al., 2022; Bülow, 2003; Swoboda, 2006). Illness dismissal is the tension that occurs when healthcare professionals question patients’ subjective symptoms and psychological status, rather than legitimising illness in the absence of causal mechanisms and objective results (Barker et al., 2022; Conrad and Barker, 2010; Roth and Gadebusch-Bondio, 2022). Evidence of illness dismissal can be seen through social constructs, such as stigmatisation, trivialisation and feminisation of reported symptoms (Bülow, 2003), leaving people trying to ‘prove’ their subjective illness experiences objectively (Dumit, 2006).

Diagnostic uncertainty has been shown through language patterns used in online forums, wherein people shared experiences of medical testing to seek or compare a diagnosis with others (Thompson et al., 2022). Test seeking allows people without a long COVID diagnosis to make sense of symptoms and experiences, in the face of inadequate support (Baz et al., 2023). An indication that an illness is becoming contested can be people systematically reporting poor symptom validation or illness dismissal within medical encounters (Bülow, 2003), and there is growing evidence about this in people seeking healthcare for long COVID (Au et al., 2022; Neaves, 2022). Research confirms people’s accounts of symptom and illness dismissal by healthcare professionals, and also indicates that this may relate to factors including (but not limited to age, gender, race, psychologised symptoms, obesity and reliance on objective testing with positive results (Harrison et al., 2024; Miyake and Martin, 2021; Moretti et al., 2022; Skilbeck et al., 2023). Some people also described feeling supported when healthcare professionals validated their symptoms, were empathetic, listened, were willing to try differing treatment options, and researched long COVID (Au et al., 2022; Harrison et al., 2024). However, if their recovery experience was poor, it led some to seek self-management practices that were poorly evidenced, ineffective and potentially unsafe (Hossain et al., 2023).

Recent explorations of long Covid experiences are situated within broader contexts of illness dismissal. A review of qualitative literature covering 13 illnesses categorised as difficult to diagnose and/or contested, identifies serious consequences of invalidation (actual or perceived) for long Covid patients (Bontempo et al., 2025). This review proposed a typology of effects that that ranged from mild feelings of self-doubt to suicidal ideation. Such effects were not limited to emotional and cognitive manifestations; they also included health care related effects such as depleted trust in clinicians and avoidance of seeking healthcare altogether. Given such potentially detrimental outcomes, the importance further investigation cannot be understated, and the imperative to make such experiences visible has also included co-produced research with long Covid advocacy groups (Clutterbuck et al., 2024). Co-developed recommendations focused on already disadvantaged communities and called for clearer public health messaging to raise the awareness of long COVID. In light of previous observed trivialisations of the condition, asserting (for example) that it was ‘the new bad luck’ (Morrison, 2023), attention is increasingly focussing on long COVIDs association with contestation and stigma – asserted and anticipated (Farrimond and Michael, 2025)

Contested illnesses are problematic in conjunction with conceptual frameworks that objectively define illness (Bülow, 2003). Therefore, exploring long COVID’s illness status requires the consideration of older theoretical frameworks, such as the sick role theory (Konsman, 2021). Parsons’s (1975) sick role theory remains a valuable and valid framework for understanding current illness experiences, chronicity and contested status, such as distinguishing a condition from an acute or chronic illness (Schipke, 2021). This is relevant as the notion of an acute illness was essentially reconceptualised during the COVID-19 pandemic. People who were asymptomatic or free from illness were positioned within a sick role by societal and illness behaviour restrictions such as lockdowns and medical access over previously being able to self-position themselves within the patient-sick role (Amoretti and Lalumera, 2020). In such circumstances, a person cannot always align with the social and behavioural norms posed by the sick role (Cheshire et al., 2021). Parsons originally outlined four sick role tenets of returning to normative societal roles after acute illness. The first was that people’s motivation to be exempt from performing a societal role was because of socially defined and validated illness. The second exempted people from any responsibility for being ill and assumed recovery to be ‘spontaneous’ or treatable. The third was that claims for exemptions from societal roles did not legitimise occupying the sick role for longer than socially defined, meaning that people were expected to resume societal roles as quickly as possible. The fourth and final tenet was that being sick qualified healthcare professionals to manage illness, while patients were assumed to cooperate and comply with the obligations to recover from illness (Parsons, 1951).

Literature of reported experiences of long COVID within the context of the sick role is emerging. After meeting objective criteria to warrant hospital discharge people were deemed clinically well. However, when assuming the patient role again with long COVID, they were being perceived differently by others as their illness had become a chronic and subjective condition without effective treatment or diagnostic certainty (Wang et al., 2022), resulting in feeling the need to leave the sick role to regain pre-COVID-19 health and identity (Skilbeck, 2023). Some reported pressure to recover and felt guilt when unable to return to pre-COVID-19 roles (Harrison et al., 2024).

Prior literature contains accounts of ME/CFS through a sick role lens to provide critical insight into recovery behaviours (Cheshire et al., 2021), however this lens has not been applied in the context of long COVID. Given its unknown causal mechanisms, diagnostic uncertainty, poor recovery/treatment outcomes and reports of illness dismissal, it is necessary to examine accounts of people’s lived experiences with long COVID to explore the contestation surrounding it, especially relating to communication and management (Au et al., 2022; Barker et al., 2022). Encouragingly, research now capitalises on online interaction, examining the benefits of shared personal expertise and navigating diagnostic processes, also arguing that online discussion forums help to define long COVID (Almqvist-Ingersoll, 2025). Our study expands on this notion by examining the positioning of functional roles in a medical encounter when people seek healthcare for long COVID under the framework of the sick role. It specifically explores the contested aspects of long COVID through qualitatively analysing online forum narrative accounts and people's experiences when seeking healthcare.

## Method

### Study design

We undertook a reflexive thematic analysis of accounts within Reddit and Mayo Clinic Connect public online discussion forums. Such forums contain lists of topics for discussion known as ‘threads’, which generate replies known as ‘posts’. (Giles, 2017). Online discussion forums allow retrospective analyses of contemporaneous naturalistic accounts of illness experiences within specific health-related topics (Jowett, 2015; Walsh et al., 2022). As there are limitations to retrospective interview studies, including participant recall and dependency on interview questions posed (Potter and Hepburn, 2005), the practical advantages of internet forum analysis, such as relative ease of access, are well documented (Giles, 2017; Walsh et al., 2022). There is also a higher likelihood of a person reporting immediate rather than retrospective experiences with limited influence by the researcher’s presence (Seale et al., 2010).

A Google keyword search located long COVID forum websites. A template analysis was used to identify forum threads due to the extensive material within both forums. Reflexive thematic analysis was used to analyse posts inductively, which were then further analysed within a positioning theory framework.

### Data sourcing

A Google search identified discussion forums containing the following terms: ‘long COVID discussion boards’, ‘long COVID forums’, and ‘long COVID discussion board forum’. The search identified ten websites containing discussion fora. Websites with inactive forums, ambiguous public access permissions to read content, no information advising users that contributions were publicly viewable, membership to view content and not specifically related to long COVID recovery, were excluded, after which two forums remained. Reddit (subreddit) ‘*r/covidlonghaulers*’ was chosen due to its global membership and specific long COVID creation date supported by reviewed literature. Research utilising Reddit has recently gained prominence across several disciplines, amongst which medicine and health are well represented (Proferes et al., 2021) The second, Mayo Clinic Connect ‘*Post-COVID Recovery & COVID-19 Support Group*’ was chosen due to its specific focus and also had global membership.

A template analysis used a set of a-priori codes to identify threads (Brooks et al., 2015; King, 2004). This was necessary due to the high volume of thread creation and associated posts, especially within Reddit. Four a-priori codes - Uncertainty, Stigma and Morality, Challenging Medicine, and Time/Self in Chronic Illness, were drawn from Bülow’s (2003) commentary on exploring chronic and contested illness. These codes provided the basis for the thread selection. For example, ‘Challenging Medicine’ led to threads where forum contributors described challenging medical expertise and informed the selection of the thread ‘*How to deal with dismissive, uncaring Drs*’. Threads were included if they were written in English and created no longer than a month prior to viewing (date range: 18/06/2023 – 18/07/2023). A short time frame was necessary due to the extensive daily thread creation and related posts. Threads were excluded if membership and password access were required to view posts, as were threads explicitly related to mental health concerns, pharmacology, research articles, or participant surveys. Twelve threads were identified for further analysis (Reddit = 6; Mayo Clinic Connect = 6). The twelve threads comprised 305 posts (Reddit = 183; Mayo Clinic Connect = 116).

### Analytic processes

#### Inductive analytic process – reflexive thematic analysis

Analysis followed Braun and Clarke’s (2021) recommendations to organise posts into themes. Reflexive thematic analysis was necessary due to the extensive nature and varied posts contained within the threads. In phase one, notes were taken while initially reading posts. Phase two involved creating initial codes with semantic (surface) and latent (conceptual) meanings. Phase three generated initial themes from identified codes, and phases four and five involved developing and refining final themes. An example of code-to-theme development is shown in Table 1.

**Table 1.** Example of code-to-theme development for ‘importance of symptom legitimacy’ additional analytic framework.

Initial codes	Provisional themes	Final theme
Seen, seeing or referral		
Recommendation/test	Symptom testing	
Test taken	Communication with healthcare professionals	The Importance of Symptom Legitimacy
Healthcare Professional information received	Healthcare professional engagement through referral	
Abnormal tests		

In addition to organising data thematically, the analysis included another interpretive layer of positioning theory (Harré, 2006). Positioning theory argues that people’s roles and positions share commonalities in that they both existed before a person occupied them. For example, the role/position of a doctor or health professional existed before a person occupied it. Positioning theory encompasses people positioning themselves (self-position) or others (other positioning) within an encounter. It is, therefore, possible to classify the online posts and positions reflected within them as first, second and third-order positions (positioning acts). The first-order position is where the post and positions remain unchallenged within the thread discussion. The second-order position sees a post and position challenged by another forum contributor’s response. The third-order position relates to a post from a previous encounter, such as an experience within a medical encounter. Positioning also involves a person's ‘rights and duties’ within the occupied position, where the person’s rights and duties can be tacit (assumed), deliberate (explicit), or moral (obliged to perform; Hirvonen, 2016).

Positioning theory can provide added meaning to online forum accounts and requires three analytical conditions: storylines, speech acts (illocutionary force), and positioning – the positioning triangle (Hirvonen, 2016). The positioning triangle is especially relevant to an online forum analysis of long COVID experiences because (a) many replies to a post are brief and assume a conversational turn, (b) secondly, the overall nature of healthcare professionals indirectly contributing to someone’s illness experience necessitates an examination of positions taken within these online accounts, and (c) situating long COVID within a position relating to societal sick role expectations remains unexplored. Table 2 provides some examples of positioning and positioning acts in our data.

**Table 2.** Examples of positioning and positioning acts.

	Explanation	Example	Analysis
Positioning			
Self-positioning	People position themselves within that encounter.	I had COVID-19 in February 2021 and lost both taste and smell. The taste came back. It is now over two years since I have not had any smell (C1M)	The member positions themself as having long COVID and the right to membership within the forum.
Positioning others	People position another or others within that encounter.	I will continue to book appointments with doctors regularly to see if they have reached the point of thinking outside the medical school box of labels (C15M)	The member positions healthcare professionals as having a duty to control and provide their long COVID care.
Positioning acts			
First order position	A position (rights and duties) remains unchallenged by another or others.	After 1.5 years, I did similar testing, came back negative (C3R)	The post remained unchallenged by other members for the member's self-positioning of not recovering from long COVID.
Second order position	Another or others challenge a position (rights and duties).	The doctor informed me that adults generally get them through ageing. I had the doctor check again this year, and no change. I am unsure due to my breath shortness and exhaustion when moving (C7M)	The member challenges their healthcare professional’s duty to provide care by dismissing their long COVID experience as age-related and beginning to re-position control of their illness as long COVID.
Third order position	A position (rights and duties) described to others from a previous encounter experienced.	The first consultation was over the phone, answering questions. I had a second consultation with a nurse who followed up with questions from my first consultation. They recorded answers into a computer where the nurse made test recommendations to address the shortness of breath upon exertion (C6M)	The post shows the member’s first-person account of a medical encounter, the positioning of the patient to receive care, and the healthcare professional’s duty to provide care and control the illness experience of long COVID.

### Quality appraisal

All authors engaged in reflexive approaches that critically assessed the analysis process, decisions made and rationale for making them (Braun and Clarke, 2021). We were mindful of our own COVID-19 experiences and recovery. We noted and regularly discussed reflective statements about why specific threads were selected and continually reviewed if they aligned with a-priori codes. Regular reacquaintance with a-priori codes informed thread selection or exclusion in conjunction with personal reflections.

### Ethical considerations

Ethical approval was obtained from the Southern Cross University Human Research Ethics Committee. In selecting the two websites for analysis, we ensured that their terms and conditions explicitly stated that forum contributors posted content with the full knowledge that their text was publicly available. We did not need password access to read the forum material, ensuring passive analysis without correspondence with online forum members. The study was granted a waiver of consent based on the Australian National Statement on Ethical Conduct in Human Research (2023). The waiver of consent related to the use of secondary data, with little to no risk of harm due to no active personal participation. Online posts were already de-identified by creating alternate usernames, so there was no practical way to gain consent. We also maximised privacy in several ways. While the online posts mainly were deidentified by contributors’ use of pseudonyms as usernames, we strictly adhered to the following additional measures: (a) excluding posts that appeared to have used a real name, mentioned other people by name (e.g., health professionals), or included media other than text (e.g. identifying photographs), (b) replacing forum usernames with contributor numbers for results reporting, (c) paraphrasing forum extracts to minimise the possibility of locating accounts through direct phrase searching and (d) managing data confidentiality by securely storing Word documents relating to threads and posts on private Southern Cross University servers.

## Results

This section presents the outcomes of the thematic analysis in conjunction with the role principles of positioning theory. Themes were not exclusive to either Reddit or Mayo Clinic Connect separately and are therefore presented from both forums simultaneously. The analysis identified three themes: ‘The concept of time’, ‘The importance of symptom legitimacy’, ‘Responding to diagnostic dismissal’. Illustrative extracts are provided to support each theme. Each extract is paraphrased to avoid locating the exact post and risking identification, with usernames replaced with contributor numbers related to each subforum, such as C1R (Contributor 1, Reddit) and C1M (Contributor 1, Mayo Clinic Connect).

### The concept of time

The contributors’ most prevalent expression of time was stating a combination of the month and year since their acute COVID-19 infection or the time they had with long COVID. This timestamping of how long they had been experiencing long COVID was commonly reported in posts across all threads.

- *I had COVID-19 in February* 2021 *and lost both taste and smell. The taste came back. It is now over two years since I have not had any smell* (C1M)
- Forty-two months since the first infection and fourteen since the second (C1R)
- I lost smell and taste over sixteen months ago. There is no control, and it is out of hand (C2M)
- I am 6 months after infection. I can just get out of bed and sit down while I shower (C3R)

The accounts reflect contributors contracting long COVID, where a timestamp is a specific reference that informs others of the length of time they have experienced poor recovery outcomes. The timestamp also shows the length of time with long COVID and having no control over symptom presentation and recovery prospects, as seen in C2M. Importantly, these posts also show that contributors placed a causal link between COVID-19 infection and the onset of enduring long COVID symptoms, as seen in C1M, C1R and C3R.

Positioning analysis of the long COVID timestamp is evidence of first-order positioning where the assertion of having long COVID remains unchallenged by others within the forum. The contributors positioned themselves as having long COVID through the timestamp that saw others within the forum not challenging poor recovery outcomes after acute COVID-19 infection. Nor did others challenge the inference that acute COVID-19 infection was the causal link for their long COVID symptom and illness experiences. Through contributors not challenging the long COVID timestamp, it then positions the contributor’s right to engage in the forum whilst also providing a basis for the acknowledgement and legitimacy of symptoms and illness experiences, thus providing support to symptom legitimacy being a motivational factor for engaging within the forum.

The second expression of time was within the contributor’s medical encounters and long COVID recovery outcomes, as shown by the following:

- *I had COVID-19 in November and December* 2020. *The taste has not returned, but the smell has. My doctor says the taste may return in time, too* (C3M)

Contributors reported that healthcare professionals stated symptoms would return to normal within an undetermined timeframe. The account is the contributor’s acknowledgement of having poor short-term recovery outcomes or effective treatment for their taste to return. It is also an initial inference of the contributor’s cognitive realisation of indefinite and ongoing symptom self-management.

Account C3M is an example of third-order positioning within a medical encounter. It shows that the healthcare professional is initially self-positioned within the moral duty to provide illness management and answers, and the contributor is self-positioned within the moral right to receive the same as a long COVID patient. However, once the healthcare professional cannot fulfil this duty through long COVID’s poor treatment options and inability to predict a recovery outcome, the contributor is re-positioned outside the moral rights and duties of the medical encounter. This is evidenced through time experienced without effective treatment and predictive recovery outcomes, as seen in C3M, and further supports the thematic cognitive realisation of ongoing self-management.

### The importance of symptom legitimacy

Contributors expressed the importance of symptom legitimacy when seeking healthcare for long COVID through healthcare professional engagement, provision of objective testing and receiving abnormal results.

- *A few doctors I have had, about eight of ten, appear to care. Those that did let me get an MRI, and another put me for assessment of ADHD even though I scored low on the doctor's test* (C4R)
- *I had low potassium, usually obtained through vomiting or diarrhea. I had neither; my diet was good, with enough potassium. The results left the doctor rubbing his head* (C5R)
- *My pathology looked red all over. I have never been so happy about getting bad results* (C4M)
- *The first consultation was over the phone, answering questions. I had a second consultation with a nurse who followed up with questions from my first consultation. They recorded answers into a computer where the nurse made test recommendations to address the shortness of breath upon exertion* (C5M)

Healthcare professionals’ engagement at the first instance, subsequent follow-up appointments and resultant symptom testing provided contributors with the legitimisation of their symptom experiences, as seen in C5M. Account C5M shows the meaningfulness placed behind healthcare professional engagement as an acknowledgement of long COVID symptoms. The contributor relays a detailed account of the interaction and places importance on communication within the encounter and receiving follow-up appointments as subjective acknowledgements of symptoms. The addition of receiving objective test recommendations further enhances subjective symptom legitimacy, providing the potential for objective answers to legitimise the illness experience further.

Contributors expressed the importance of symptom legitimacy through not only healthcare professional engagement and the provision of diagnostic testing but also receiving abnormal results, as seen in C4R. Diagnostic testing provided a gateway to objectively acknowledging the subjective reality of their long COVID symptoms, as seen in C4M, who expressed this objective reality and legitimacy of symptoms through the elation felt when receiving abnormal results despite not being provided with a formal diagnosis of long COVID, as seen in C5R.

The accounts predominantly used third-order positioning, where contributors saw the healthcare professional seeming to comply with the moral duty to acknowledge symptomology and attempt to answer long COVID symptoms, as seen in C5M. Account C4R further supports this positional importance by acknowledging that those who engaged in the medical encounters and provided objective testing were seen to fulfil their role and cared about their patients’ long COVID experiences over those who did not. Thus, additional support is provided to the thematic importance of subjective symptom acknowledgement and legitimacy through engagement within the medical encounter and testing without needing a long COVID diagnosis.

### Responding to diagnostic dismissal

Forum posts contained contributors’ accounts responding to a healthcare professional’s long COVID diagnostic dismissal. These responses saw contributors challenge alternate diagnoses by sharing them with others through self-advocacy of their healthcare. The following accounts exemplify this:

- *The doctor informed me that adults generally get [symptoms] through ageing. I had the doctor check again this year and no change. I am sceptical due to my breath shortness and exhaustion when moving* (C6M)
- *The doctor said I look normal, and that is upsetting and hard to hear* (C7M)
- *The allied health professional insists it does not appear to be [long COVID]. Theorising it is something viral or in response to trauma, and I need to take a break as I have some type of PTSD* (C6R)
- *Being labelled with a panic disorder follows me like a bright sign that tells doctors not to take my concerns seriously* (C7R)
- *My doctor knows nothing about long COVID, yet is ready to provide dim predictions* (C8R)
- *I have experienced anxiety and depression for 27 years. I know the blasted difference* (C9R)

C6M shows the cognitive process of beginning to challenge a health professional with an alternate diagnosis of it being age-related rather than long COVID for breathing and fatigue difficulties. The predominant dismissal reported by contributors was having their long COVID experiences psychologised, identifying PTSD, panic disorder, anxiety and depression, as seen in accounts C6R, C7R, C8R, and C9R. Receiving such diagnoses also saw contributors express ongoing stigmatisation when seeking care for long COVID, as seen in C7R. The impact of receiving an alternate diagnosis or the appearance of looking normal yielded expressions of disappointment, distress, and anger, as seen in C7M and C9R. Within the account, C7M shows the healthcare professional's reliance on objective results to provide a long COVID diagnosis.

The accounts show third-order positioning, whereby the contributors challenge healthcare professionals’ failure to acknowledge and validate the illness experience of long COVID. The diagnostic dismissals see the removal of the contributor’s rights and duties and re-positioned outside the confines of the medical encounter management through the reported avoidance of, or reluctance to, providing a long COVID diagnosis. In challenging the diagnostic dismissal, contributors re-position themselves back within the right to long COVID legitimacy and subsequent motivating factors for engaging within the forum. The accounts also evidence the first-order positioning that remained unchallenged by other contributors, thus receiving illness validation through engagement with others and providing additional supportive evidence for seeking validation after dismissal within online forums.

The following accounts represent examples of contributors challenging diagnostic dismissal through self-advocacy.

- *I see it as an elimination process. Internet search for each symptom and find the specialist who manages it. Get them to order the required tests, so you know it is not life-threatening. Repeat each symptom to give you some peace of mind* (C8M)
- *I have been trying to manage long COVID for 1 year. I am determined to advocate for myself by requesting tests, supplements and anything that may help me* (C9M)
- *I found a fatigue centre that accepts my insurance. If you live near there, it might be worth a go. I am going to go there* (C10M)
- *I will continue to book appointments with doctors regularly to see if they have reached the point of thinking outside the medical school box of labels* (C11M)
- *People need to begin demanding evidence for every claim and diagnosis the doctor puts onto paperwork* (C10R)

Contributors challenge the rights and duties held within the medical encounter after experiencing dismissal through self-advocacy. Self-advocacy shows contributors’ responses to dismissal by seeking healthcare professionals to legitimise long COVID or to account for an alternate diagnosis, as seen in C10R. The accounts represent individual and collective advocacy, where contributors encourage others to seek specialist healthcare options that manage fatigue, as shown in accounts C8M and C10M. Self-advocacy is also seen through repeated test-seeking efforts to legitimise symptoms after dismissal and control their illness experience, as seen in C8M, by providing the contributor with ‘peace of mind’.

Further evidence of self-advocacy providing a sense of control is seen in C9M, where the contributor highlights test-seeking and finding any beneficial treatment in response to lengthy self-management of symptoms. Self-advocacy creates an opportunity to provide an objective reality and meaning after dismissing the subjective presentation of symptoms, as evidenced by contributors C8M, C11M, and C10R. The contributors actively self-advocate to re-position themselves and others within the medical encounter and the moral right to have symptom recognition and illness legitimacy. In doing so, they do not accept healthcare professional dismissals, such as receiving an alternate diagnosis, as seen in C10R. Contributors further challenge dismissal by regaining control over their illness experience, as seen in C9M, where prolonged self-management sees the cognitive shift of the contributor begin to regain control over their illness experience through re-positioning themselves within the medical encounter.

## Discussion

### Summary of findings

The thematic analysis resulted in three themes: ‘Concept of time’, ‘Importance of symptom legitimacy’, ‘Responding to diagnostic dismissal’. Further analysis using positioning theory showed the perceived incapacity of healthcare professionals to provide effective treatment, predict recovery outcomes, and the decisions behind dismissing people's long COVID experiences. This supported the thematic results and long COVID being situated as a contested illness despite scientific research advances in its biomedical trajectory.

### Relationship to previous research

‘The concept of time’ theme, referenced by the online declaration of a timestamp, provided interpretations supporting long COVID’s contested nature. The expression of time since infection, or time experienced with long COVID referencing poor recovery outcomes inferred chronicity. Time referenced in this way further supports previous language pattern analysis of long online COVID forum accounts and attributing long COVID chronicity to poor recovery outcomes (Thompson et al., 2022). The long COVID timestamp was also an indirect inference that COVID-19 was the causal mechanism behind long COVID symptom experiences, through reporting time from infection to time without recovery, which supports current long COVID immunological (Altmann et al., 2023), and qualitative long COVID contested illness findings (Au et al., 2022).

Previous reports of people experiencing dismissal when seeking a long COVID diagnosis and subsequent care indicate a possible contested status (Au et al., 2022; Harrison et al., 2024; Hossain et al., 2023). Evidence of dismissal was thematically evident in our data in two ways. The first was through members challenging dismissal through posting experiences within online forums. Dismissal experiences shared online followed receiving psychologised diagnoses such as PTSD, anxiety, depression, and panic disorder. Other reported experiences were when members received alternative explanations such as age-related, relying on objective testing giving abnormal results, and looking ‘normal’. These findings further support the reasoning behind the patient-led definition of long COVID that challenged public health messaging and diagnostic dismissal experiences at the beginning and throughout the pandemic (Au et al., 2022; Callard and Perego, 2021; Perego et al., 2020; Miyake and Martin, 2021; Harrison et al., 2024).

The second challenging response was to attempt to prove their experiences through self-advocacy. The self-advocating behaviours included seeking control through objective tests to obtain objective legitimacy, finding effective treatment, seeking alternative specialist healthcare options to provide supportive management or acknowledgement, and seeking answers to provide an alternative diagnosis. Such findings continue to support prior experiential long COVID literature that a health professional’s inability or reluctance to diagnose long COVID without objective certainty (Au et al., 2022; Barker et al., 2022; Harrison et al., 2024) or inability to manage over time (Bülow, 2003) leaves people in the position to self-advocate or the need to ‘prove’ their illness experience (Dumit, 2006). Self-advocacy was a member's gateway to recovery through objective tests, finding an effective treatment for symptoms, and providing answers to an alternate diagnosis support findings that advocacy allowed people with long COVID to seek answers (Baz et al., 2023).

Positioning theory provided a further analytic framework that facilitated additional interpretation of the identified themes (Harré, 2006). It fostered deeper consideration of the moral order of social actions, the rights and duties of an encounter, and why people act and speak in a certain way in the specific context of the long COVID experience. Positioning analysis saw dysfunction and function between moral rights and duties when people sought care for long COVID. Dysfunction was evident when people were re-positioned by the healthcare professional outside the moral rights and duties held within the medical encounter. This re-positioning occurred when the healthcare professional could not predict a recovery outcome, provide effective treatment, or dismiss long COVID with an alternate diagnosis due to causal and diagnostic uncertainty. In contrast, positional moral rights and duties remained functional when the healthcare professional legitimised symptoms through subjective acknowledgement, the objective provision of symptom testing—with or without abnormal results providing a long COVID diagnosis, thus providing additional support to existing arguments about seeking legitimacy as a motivational factor when seeking health care (Brehon et al., 2023). However, when a person was re-positioned outside the medical encounter and from the care and management of the healthcare professional, forum contributors used positional speech and acts to challenge or accept the healthcare professional’s inability or reluctance to diagnose or manage long COVID.

Additionally, the positioning analysis showed why people remained within the management of the health care professional when their long COVID symptom presentation was acknowledged through subjective engagement, objective testing and receiving abnormal results. This acknowledgement supports the importance placed on symptom legitimacy (Au et al., 2022; Harrison et al., 2024). Diagnostic ability is a key determinant of contested illness as formal diagnosis leads to social recognition and acceptance (Dumit, 2006).

The sick role applies explicitly to the sociological examination of patient roles and illness behaviours within a medical encounter (Schipke, 2021), primarily for a person to recover mentally or physically, and where the therapeutic interventions restore a person to normative societal functions (Parsons, 1951). Applying the sick role theory provides a specific theoretical premise for studying long COVIDs chronic nature (Schipke, 2021). A person’s inability to comply with the behavioural norm and illness behaviours of the sick role indicates that an illness is chronic (Konsman, 2021; Parsons, 1951; Schipke, 2021) and, as this study explores, the nature of illness behaviours associated with long COVID being contested.

Positional analysis provided two primary reasons for people seeking healthcare for long COVID. The first was to seek assistance in making a full recovery, and the other was to seek legitimacy for symptoms or illness experiences. While it can be argued that the motivational factors within this study showed that people were not necessarily motivated to exclude themselves from societal roles, their attempt to find assistance for recovery outcomes and seek symptom legitimacy is confounded by unknown causal links, diagnostic uncertainty, poor recovery/treatment outcomes that are characteristics of contested illnesses such as ME/CFS (Bülow, 2003; Swoboda, 2006). Furthermore, the perceived legitimacy of long COVID was aligned to engagement with healthcare services, placing emphasis on testing and thereby validating symptoms. This is broadly commensurate with previous observations of documenting recovery, in which long COVID patients believed their experiences to be perceived as inauthentic compared to those who had experienced acute ICU admissions for COVID (Maclean et al., 2025)

Time since infection without recovery, healthcare professionals’ inability to explain causes, diagnostic uncertainty, predict recovery, poor treatment efficacy and evidence of symptom and illness dismissal shows that a person with long COVID currently has limited ability to be exempt from performing a societal role by a socially defined and validated illness. Therefore, a person with long COVID is unlikely to align with the sick role illness expectations, which further supports long COVID’s chronic nature and possible categorisation as a contested illness (Au et al., 2022; Barker et al., 2022; Neaves, 2022).

Positioning theory furthers this contested trajectory within the sick role as it repeatedly shows people actively transition into the patient role within the medical encounter, further supporting prior long COVID literature when seeking the patient role after acute COVID-19 recovery (Wang et al., 2022). The study’s findings support prior thematic interpretations that people were motivated to leave the sick role to regain their pre-COVID-19 health and identity (Skilbeck, 2023; Wang et al., 2022).

Positioning analysis provides an additional analytic layer where online accounts of third-order positioning revealed a misalignment between the healthcare professionals’ moral rights and duties and patients’ rights and duties to receive management and care - posed by the complexities of long COVID diagnostic uncertainty and heterogeneous presentation. As with many illnesses, people commonly get sick and recover; however, some people with long COVID are not recovering (Bonsaksen et al., 2022). The patient role is the foundation of the sick role from which all healthcare professional roles are derived and valid for understanding the dichotomy between patient and healthcare professional and the effect on the structural roles and relationships when exploring patient experiences subjectively (Schipke, 2021). However, with the distance of time since the pandemic declaration and the continued reporting of poor recovery outcomes and treatment options, a long COVID status is open to contestation. This contested illness trajectory is further supported by its continued unknown causal mechanisms, diagnostic certainty and the reported reluctance of healthcare professionals to legitimise symptom and illness experiences without objective certainty (Bülow, 2003; Conrad and Barker, 2010; Swoboda, 2006).

Considering long COVID experience in the context of positioning theory extends beyond the reported interactions here, and research should endeavour to situate the competing knowledges of this complex condition to increase perceptions of legitimacy in broader psychological and societal contexts. The presence of online discussions and associated activism helps to make these positions visible, further strengthening calls for recognition as digital footprints increase. As Farrimond and Michael (2025) proposed, it is useful to think of long Covid as occupying multiple ontologies and strengthens the utility of deploying positioning theory as a framework for interrogating difference and contestation relating to an illness that is both widespread, yet relatively novel.

### Limitations and future directions

While online data analysis does not equate to that drawn from face-to-face interviews, it allows the exploration of contemporaneous naturalistic accounts without the researcher's influence (Walsh et al., 2022). As forum contributors posted anonymously, this study cannot infer contributor demographics or broader cultural/health system differences. However, data were obtained from subforums with globally accessible membership free from financial costs to engage in the forums. Therefore, it can be assumed that contributors’ engagement was drawn from differing backgrounds and cultural contexts. Furthermore, the analysis focused on the thematic and positional content of online long COVID encounters, which did not require structural comparisons between contributors’ characteristics and environmental contexts. The data collection was small but necessary due to the rapidly expanding daily additions to forums. But it is important to consider that the online posts were published three years after the initial declaration of the pandemic. By this time, some health professionals have reasonably had greater understanding of long COVID management, which may not be represented in this data sample. Unless a post contained a specific declaration of when an interaction occurred it is not always possible to know this information However, the final number of posts identified for analysis allowed for interpretations of long COVID experiences (Braun and Clarke, 2022) that triangulated data from two long COVID forums (Hanson et al., 2005).

Although exploratory, these results support limited experiential research into the contested nature of long COVID and provide future directions for continued research. Further research would benefit from obtaining healthcare professionals’ perspectives on clinical discussions and managing the condition. Additionally, future analyses could focus on exploring the motivations behind seeking healthcare, cultural factors influencing long COVID diagnostic dismissal, and societal influences behind illness behaviours when seeking recovery. More extensive attention on exploring the clinical positional rights and duties within the sick role framework will continue to provide data on limited research into long COVID’s contested associations. Prior research provides evidence of people’s emotional responses to their long COVID experiences, but an exploration of emotional responses was not within the scope of this study. Future work could assess the emotional content of forum posts in more detail to examine the affective consequences of seeking healthcare for long COVID.

## Conclusion

The analysis of online forums allowed a contemporaneous view of current global experiences assessing long COVID’s contested nature. The study shows individual experiences and perceptions of what they believed to be healthcare professionals re-positioning them outside the moral rights and duties of the medical encounter. This extended to assumptions of non-compliance relating to sick role behavioural norms that support long COVID’s trajectory to becoming a contested illness. The study highlights the need for a continued understanding of its contested nature from long COVID patients and those morally positioned to provide management and care. It shows the importance of healthcare professional engagement and support measures to prevent self-management of symptoms outside clinical interactions. Long COVID holds global economic and quality-of-life ramifications in managing health policies, practices and individuals who continue navigating the complexity surrounding long COVID. The estimated number of people with long COVID continues to rise, and it is, therefore, necessary to continue gathering intelligence about their experiences when seeking care.
